# Neurovascular coupling during hypercapnia in cerebral blood flow regulation

**DOI:** 10.1038/s41467-024-50165-8

**Published:** 2024-09-02

**Authors:** Grant R. Gordon

**Affiliations:** Hotchkiss Brain Institute, Department of Physiology and Pharmacology, Cumming School of Medicine, University of Calgary, Calgary, AB T2N 4N1 Canada

**Keywords:** Neuro-vascular interactions, Neurology

## Abstract

Neuronal activity consumes cellular energy and generates carbon dioxide (CO_2_). To counter this metabolic challenge, synaptic signalling communicates with nearby microvasculature to increase local blood flow. Is this process solely based on feedforward synaptic signalling, or is the generated CO_2_ also involved? This question was addressed in mice in a new Nature Communications publication by Tournissac and colleagues where they showed that neurovascular coupling is not affected by exogenous CO_2_ or its associated acidification.

Functional hyperemia is a type of neurovascular coupling, whereby enhanced neuronal activity initiates an increase in local cerebral blood flow to deliver more oxygen and glucose to working brain cells. This is a robust and reliable brain process that is the basis of several functional imaging techniques. Carbon dioxide (CO_2_) is equally, if not more, robust at increasing brain blood flow, in a process called hypercapnic hyperemia. Systemic hypercapnia, caused by breathing high CO_2_, breath holding, or many medical disorders, causes a global increase in brain blood flow when CO_2_ reaches the brain. As neuronal activity drives high rates of oxidative phosphorylation producing CO_2_, an important question is whether functional hyperemia makes use of metabolically elevated CO_2_ to increase local brain blood flow?

This is the topic of a new paper by Tournissac et al.^1^ published in *Nature Communications*. Another study on the same topic was published two years earlier in the same journal^2^. There, Hosford and colleagues showed that breathing elevated CO_2_ occluded functional hyperemia caused by sensory stimulation. The idea was to increase CO_2_ in the systemic blood supply and saturate any local CO_2_ control systems in the brain. The main method used to quantify cerebral blood perfusion was functional magnetic resonance imaging (fMRI). The conclusion was that hypercapnic hyperemia and functional hyperemia converge on the same cellular pathways, and that functional hyperemia relies on local CO_2_ generation to increase cerebral blood flow. In contrast, this new paper by Tournissac and colleagues uses a different approach with two-photon fluorescence vascular imaging and functional ultrasound but with the same technique to elevate brain CO_2_. They showed that the two types of cerebral blood flow responses were completely additive i.e. they did not occlude each other. The authors concluded that hypercapnic hyperemia and functional hyperemia operate through separate cellular pathways. Before we delve further into both papers, first some reflections.

Historically, functional hyperemia was viewed as a metabolic negative feedback loop^3^. Here, neuronal activation is met with the consumption of ATP and adenosine generation, drops in local oxygen and the production of CO_2_ to generate new ATP via oxidative phosphorylation. CO_2_ generation decreases pH due to the bidirectional reaction between CO2 + H_2_O and HCO_3_^−^ + H^+^ which is facilitated by carbonic anhydrase. CO_2_, together with H^+^, is a potent vasodilator. Above a specific threshold, every 5 mmHg of CO_2_ elevation in arterial blood is met with a 50% increase in arterial blood velocity in humans^4^. Early on, CO_2_, along with adenosine and oxygen, were suspected to dilate the cerebral microvasculature, and once new energy substrates were replenished, homeostasis would be restored and cerebral blood flow would return to normal levels. However, work over the past 20 years has reshaped our understanding of neurovascular coupling. Our current model favours a feed-forward system, whereby glutamate synaptic signalling itself (and not the metabolic byproducts of its occurrence) initiates intracellular calcium-dependent cell cascades that generate vasoactive messengers that diffuse from synapses and glial processes to vascular contractile cells to dilate microvasculature and increase blood flow^5^. Nature may have favoured such as system because glutamate signalling is energy intensive^6^. Perhaps it is faster and more efficient to signal directly to blood vessels, as opposed to waiting for a negative feedback system to work. However, the brain may not save time by ignoring metabolic by products. Functional hyperemia takes approximately 1 sec to manifest in vivo, which is plenty of time for pH to change and metabolites to rise in the extracellular parenchyma from enhanced neuronal activity^7^. Furthermore, such metabolites could act independent of the feedforward glutamate system, or be intertwined within that signalling network. Arguing for independent action, experiments blocking multiple feedforward neurovascular coupling pathways cannot completely eliminate functional hyperemia^8,9^, suggesting other, possibly metabolic pathways, have a role in neurovascular coupling. Arguing for intertwined mechanisms, multiple blockers of the feedforward glutamate-mediated system, such as nitric oxide synthase and cyclooxygenases antagonists, also block hypercapnia hyperemia^10,11^. This raises the questions, do functional and hypercapnic hyperemia mechanisms converge on the same cellular pathways? When functional hyperemia occurs in the brain, is local CO_2_ production and its action on cells in the neurovascular unit a key mechanism in neurovascular coupling? These questions were not carefully address in rodents in vivo, until Tournissac et al. and Hosford et al. Sparse evidence from humans looking at the middle cerebral artery with transcranial doppler shows a similar effect to Hosford, that in the presence of hypercapnia, motor movement induced functional hyperemia is compromised^12^, but this study provided no controls for ceiling effects. Though Tournissac and colleagues and Hosford and colleagues came to opposite conclusions, we need to take a closer look.

In Tournissac et al. they employed continuous 10% or 10 sec of 20% CO_2_ inhalation and a combination of two-photon imaging of microcirculatory blood flow in the barrel cortex or functional ultrasound for both regional and whole brain measurements. The authors examined arteriole diameter and RBC velocity at different points in the vascular tree. They also measured intravascular pH changes to the CO_2_ challenge and intracellular calcium signals in different cell types of the neurovascular unit. They showed that brief, high CO_2_ exposure leads to reversible acidification of the blood plasma and all cells in the neurovascular unit occurring 3–4 seconds prior to a transient arteriole dilation (hypercapnic hyperemia). The authors used this time lag to study if functional hyperemia, triggered by whisker stimulation in mice, is affected by either the early CO_2_ induced acidification, or by the hypercapnic hyperemia itself. They found that neither the acidification, nor CO_2_-mediated arteriole dilation, affected the amplitude of functional hyperemia. A strength of the work was a detailed look at the timing of all these events and the experimental alignment of their occurrences to test if the responses were additive or not. In addition to these timing experiments, they also show that in the presence of continual hypercapnia, capillary RBC movements and regional blood velocity increases in response to whisker stimulation are largely unaffected. Overall, a convincing study showing that functional and hypercapnic hyperemia operate independently from one another, but what did the previous work show?

In the Hosford et al. paper, cerebral blood perfusion was measured with fMRI in rats. Arterial spin labelling was used to measure quantitative global and regional blood perfusion of the brain, and the blood-oxygen-level-dependent (BOLD) response was employed to detect relative changes in the oxy/de-oxy hemoglobin ratio which increases in the somatosensory cortex when blood vessels dilated to functional stimulation. Electrical forepaw stimulation was used to evoke neurovascular coupling. Inspiring elevated CO_2_ increased global brain blood flow, and 10% CO_2_ nearly completely occluded functional hyperemia. The authors then showed that functional hyperemia depended on the sodium bicarbonate co-transporter (NCBe1) in astrocytes using a cre-lox knockout. Due to the equilibrium between CO_2_ and bicarbonate, this transporter was an interesting target to explore. As with the Tournissac et al. work, the Hosford et al. study used state-of-the-art approaches, and was well controlled. How was this opposite result achieved?

While both impressive papers, proving that two pathways do or do not interact through an occlusion test is challenging because one or both pathways need to be near maximally activated. For example, even if functional and hypercapnic hyperemia converge on a common cell pathway, if each is only activating that pathway to 25% of maximal, then it should be expected that both together would generate a 50% response—no occlusion. Tournissac et al. appears to use a submaximal neurovascular coupling response with a relatively strong (but still likely not maximal), hypercapnic response. Thus, it is unclear if occlusion is possible to observe under these conditions. Nevertheless, their results clearly show that the two pathways do not interact at the chosen level of their stimulations. Yet, using maximal responses is also difficult as one needs to control for ceiling effects. A 100% of maximal CO_2_-mediated dilation may leave little room for further dilation to other stimuli, and in such a scenario one cannot conclude that two pathways are apart of a shared mechanism. Hosford et al. attempted to tackle these issues. To control for a blood flow ceiling effect in high CO_2_, the authors provided stronger forepaw stimulation to show that blood flow can still be partially boosted. Separately, they administered systemic vasoconstrictors, which brought cerebral blood perfusion back down, yet functional hyperemia to moderate or strong sensory stimulation was still largely occluded during hypercapnia. Furthermore, the vasoconstrictors did not block functional hyperemia in normocapnic conditions. This latter result though was surprising, that the constrictor indomethacin, which blocks cyclooxygenase and has been reported to reduce functional hyperemia^13^, did not impact sensory induced fMRI signals in their hands.

The primary measurements employed in each paper are different. While both are related to cerebral blood perfusion, the relative and quantitative fMRI techniques used by Hosford have relatively low spatiotemporal resolution and low SNR. Tournissac et al. instead measured arteriole diameter and RBC movements with 2-photon and blood velocity with functional ultrasound. Could it be that measuring different blood flow parameters is the reason for one study seeing occlusion of functional and hypercapnic hyperemia and the other not? While there is a direct relationship between all these measurement modalities and cerebral blood perfusion^14^, BOLD imaging is less sensitive at detecting changes compared to functional ultrasound and two-photon imaging^14^. Thus, it is likely that the Hosford and colleagues’ protocol would inherently require more intense and longer duration types of sensory stimulation. Given the arguments above that near maximal pathway activation may be necessary to see an occlusion between two processes, Hosford and colleagues’ experiments may be able to see this interaction, whereas Tournissac and colleagues’ not. But is Hosford and colleagues’ observation physiologically relevant? It remains unclear.

In Hosford et al., unnatural, electrical forepaw stimulation is used. While this method was confirmed to activate sensory Aβ fibers by observing repeatable synaptic field potentials and fMRI signals in the forepaw sensory region, the activation of other ascending fibers was not ruled out and they may have been recruited^15^. Either way, a more natural type of sensory stimulus is needed to better understand if/how functional and hypercapnic hyperemic responses interact. In Tournissac et al. the authors show that the neuronal GCaMP signal driving neurovascular coupling is not different in the presence of CO_2_ acidification – an important control. However, GECI fluorescence is highly sensitive to pH, which affects the dynamic range of the sensor^16^, making this control less clear. Elevating brain CO_2_ is expected to decrease neuronal excitability^2,17^, which is a issue for both papers. In Tournissac et al.’s case, one can ask why does CO_2_ not affect functional hyperemia strictly due to synaptic depression? In Hosford’s case, one can ask if this explains their main result because whenever there is high CO_2_ in their experiments, functional hyperemia is occluded (though the authors did rule out the involvement of decreased pH itself). Furthermore, to perform the in vivo experiments, Tournissac et al. employed the sedative dexmedetomidine, whereas Hosford et al. used α-chloralose anesthetic. This is suboptimal as all anesthetics/sedatives affect cerebral blood flow regulation in some way, either by acting directly on vascular control pathways, or indirectly via changes to neuronal activity and/or brain metabolism^18^. A future approach could be the use of miniscopes to measure microcirculatory changes in a freely behaving animal^19^, while in a gas-controlled chamber. This should be technically feasible and would be an interesting experimental platform to test the hypothesis.

Tournissac et al. did not explore cellular mechanisms and the author’s conclusions would be even stronger if, for example, hypercapnic vasodilation could be reduced or eliminated by blocking a specific signalling pathway, which then had no effect on functional hyperemia. While we know that NOS and COX blockers affect both responses, it would take the uncovering of new mechanisms to clarify this idea. Here, Hosford et al. demonstrated a CO_2_-related mechanism in astrocytes mediating functional hyperemia via the NCBe1. However, this was tested in an atypical neurovascular coupling preparation – a direct, invasive tissue oxygen measurement using fast cyclic voltammetry. It is necessary that the NCBe1 target be tested with other measurement techniques, such as fMRI and the methods used by Tournissac et al.

## Concluding remarks

The field needs to decide on how to further test the hypothesis. For a start, anesthetics/sedatives should be harmonized or eliminated, and a range of natural sensory stimulation intensities and durations must be tested (e.g., Tournissac and colleagues use stronger/longer stimulations, while Hosford and colleagues using weaker/shorter stimulations), and at least one micro and one meso/macro imaging modality (e.g. 2-photon and fMRI) should be employed on the same mouse. Resolving this will be important to improve our understanding of cerebral blood flow regulation, and to provide a framework for how to interpret combined functional and hypercapnic stimulations in the lab or the clinic using functional imaging techniques.
